# Revisiting Electrochemical Biosensing in the 21st Century Society for Inflammatory Cytokines Involved in Autoimmune, Neurodegenerative, Cardiac, Viral and Cancer Diseases

**DOI:** 10.3390/s21010189

**Published:** 2020-12-30

**Authors:** Susana Campuzano, Paloma Yáñez-Sedeño, José Manuel Pingarrón

**Affiliations:** Departamento de Química Analítica, Facultad de Ciencias Químicas, Universidad Complutense de Madrid, E-28040 Madrid, Spain; pingarro@quim.ucm.es

**Keywords:** inflammatory cytokines, electrochemical biosensing, nanomaterials, magnetic particles, diazonium salt chemistry, antibiofouling

## Abstract

The multifaceted key roles of cytokines in immunity and inflammatory processes have led to a high clinical interest for the determination of these biomolecules to be used as a tool in the diagnosis, prognosis, monitoring and treatment of several diseases of great current relevance (autoimmune, neurodegenerative, cardiac, viral and cancer diseases, hypercholesterolemia and diabetes). Therefore, the rapid and accurate determination of cytokine biomarkers in body fluids, cells and tissues has attracted considerable attention. However, many currently available techniques used for this purpose, although sensitive and selective, require expensive equipment and advanced human skills and do not meet the demands of today’s clinic in terms of test time, simplicity and point-of-care applicability. In the course of ongoing pursuit of new analytical methodologies, electrochemical biosensing is steadily gaining ground as a strategy suitable to develop simple, low-cost methods, with the ability for multiplexed and multiomics determinations in a short time and requiring a small amount of sample. This review article puts forward electrochemical biosensing methods reported in the last five years for the determination of cytokines, summarizes recent developments and trends through a comprehensive discussion of selected strategies, and highlights the challenges to solve in this field. Considering the key role demonstrated in the last years by different materials (with nano or micrometric size and with or without magnetic properties), in the design of analytical performance-enhanced electrochemical biosensing strategies, special attention is paid to the methods exploiting these approaches.

## 1. Introduction

Cytokines are soluble low-molecular-weight proteins secreted by cells (both immune and non-immune) responsible for regulation of host defense, tissue homeostasis, cell-to-cell communication, and inflammatory reactions. Cytokines are key biomolecules acting as mediators and modulators of the complex functional interactions and responses of the immune system [1].

There is growing evidence that the quantification of cytokines allows immune and inflammatory responses to be predicted or monitored in a comprehensive and timely manner, thus providing clinically and immunologically useful information for the diagnosis and stratification of infectious, cancer and autoimmune diseases. Owing to the highly networked nature of their functions, multiplexed detection of different cytokines in a single sample is highly desirable to obtain a more complete and accurate picture of the process [1]. This, together with the current demands of society and clinic for simple, affordable, short-time testing and application protocols at the point of care, makes it necessary to develop methodologies alternative to those currently available, which, although sensitive and selective, are not able to fully respond such needs. In this context, the great progress experienced by electrochemical biosensing in the determination of clinically relevant biomarkers (including cytokines), assisted by the use of different materials and attractive chemistries, leads one to think that things are going in the right direction. Considering this background, this review provides an overview of the state of the art, the latest advances, the potential and the versatility that electrochemical biosensing offers for the determination of cytokines. The following sections discuss the considered relevant aspects of this family of proteins, the conventional methods versus the electrochemical biosensing strategies reported for their determination in the past five years and the challenges to be faced in future perspectives.

## 2. Cytokines: Generalities, Classification, Function and Clinical Relevance

Cytokines are small bioactive proteins (~6–70 kDa), secreted by immune and non-immune cells, which are considered core indicators of the functional status of the body. They are strongly associated with the immune system acting as signaling molecules at picomolar or nanomolar concentrations to regulate inflammation and modulate cellular activities [2]. They affect almost every biological process: embryonic development, disease pathogenesis, non-specific response to infection, specific response to antigen, changes in cognitive functions and progression of the degenerative processes of aging [3].

These proteins act in concert with specific cytokine inhibitors and soluble cytokine receptors to regulate the human immune response. The binding of a cytokine ligand to its cognate receptor results in the activation of the receptor, which in turn triggers a cascade of signaling events that regulate various cellular functions, such as cell adhesion, phagocytosis, cytokine secretion, cell activation, cell proliferation, cell survival and cell death, apoptosis, angiogenesis and proliferation [4]. Cytokines make up an exceptionally large and diverse group of pro- or anti-inflammatory factors, acting as regulators of host responses to infection, immune responses, inflammation and trauma. They are grouped into families according to their structural homology or that of their receptors [4]. Cytokines are released by cells and affect the behavior of other cells, and sometimes the releasing cell itself. The term “cytokine” encompasses interleukins (ILs), chemokines, interferons, mesenchymal growth factors, the family of tumor necrosis factors and adipokines (ADPs) [3,5]. Table 1 summarizes the main functions and members of each family.

Chemokines, or “chemotactic cytokines,” make up a group of secreted proteins (<40 kDa) which induce cell migration and include IL-8, MCP-1, MIP-1α, CC and CXC. They are involved in leukocyte chemoattraction and trafficking of immune cells to locations throughout the body. Chemokines are classified in: (i) homeostatic chemokines, which are involved in immune surveillance and navigation of cells through hematopoiesis, and are typically expressed constitutively; (ii) inflammatory chemokines, which are produced during infections or as a response to an inflammatory stimulus and facilitate an immune response by targeting cells of the innate and adaptive immune system [4].

Adipokines is a group of cytokines produced from white adipose tissue, including resident macrophages in fat, with adiponectin (APN) as the most abundant adipocyte product that plays a central role in the regulation of insulin resistance [3]. APN is proposed as a biomarker of metabolic syndromes [6].

Cytokines are produced as a response to various stimuli under both pathological and physiological conditions by a broad range of cells, including immune cells like macrophages, B lymphocytes, T lymphocytes and mast cells, as well as endothelial cells, fibroblasts and various stromal cells. A given cytokine may be produced by more than one type of cell [7,8,9]. The release of pro-inflammatory cytokines leads to the activation and production of immune cells as well as to the release of further cytokines. Recent research suggests that a simultaneous release of pro- and anti-inflammatory cytokines is mandatory in any immune response [10].

There is a great clinical interest in the determination of cytokines, since elevated concentrations are associated with inflammatory disorders which underlie a vast variety of relevant diseases, including autoimmune, neurological, cancer, inflammatory (such as rheumatoid arthritis, RA, psoriasis and Crohn’s diseases) and new infection diseases. During infection, the cytokine “storm” subsides as the infection is eliminated and the genes return to their normal state of repression by histone acetylases. When cytokines genes fail to shut down, their products drive the host into a state of chronically activated cells (auto-reactive T cells), which persists and fails to die [3]. Therefore, there is increasing evidence that quantification of cytokine-based immune fingerprints provides an accurate way to characterize the immune function and to stratify and diagnose autoimmune diseases, infection, cancer and other immune-related deficiencies, as well as to monitor their evolution and treatments [1,10]. However, since the expected levels of cytokines in the extracellular milieu are at a pM concentration range, their determination requires the availability of highly sensitive analytical methods, able to perform the determination in a small number of samples to also fit children and infants [1,11]. Moreover, being aware of the dynamic transition of the immune status of sepsis patients from an initial pro-inflammatory phase (“cytokine storm”) to an anti-inflammatory phase within a short period of time (several hours to a few days), the development of accurate methodologies able to provide near real-time data of cytokines patients’ status is urgently needed to overcome the highly heterogeneous patient cohorts during the course of disease development and improve the therapy efficiency through personalized selection [1]. Therefore, the ideal detection of cytokines in the common critical care environment must meet stringent requirements, such as a sensitivity that reaches the level of a few pg mL^−1^, a sampling-to-response time of <30 min (taking into account that the immune status may change within a few hours) and the capacity for multiplexing to detect a wide variety of cytokine species in serum [1].

### Inflammatory Cytokines: Relevance and Role in Neurological, Heart and Cancer Diseases

A particularly relevant group of cytokines is that of inflammatory cytokines, due to their involvement in neurological, heart, autoimmune and cancer diseases. Systemic inflammatory disorders, resulting from infection, trauma, surgery and severe disease conditions, pose serious threats to human health leading to organ dysfunction, organ failure and mortality [1]. Inflammation is characterized in the acute phase by increased blood flow and vascular permeability along with the accumulation of fluid, leukocytes and inflammatory mediators such as cytokines. Although inflammation may induce beneficial effects, such as pathogen clearance and phagocytosis of debris and apoptotic cells besides tissue repair processes, uncontrolled inflammation can result in detrimental outcomes by producing neurotoxic factors that exacerbate neurodegenerative pathology. Furthermore, anti-inflammatory responses are regulated by proteins that inhibit signal transduction pathways, such as suppressors of cytokine signaling proteins, transcriptional repressors and anti-inflammatory molecules that help control excessive inflammation.

Inflammatory cytokines can be divided into two groups: those involved in acute inflammation and those responsible for chronic inflammation. Several cytokines play key roles in mediating acute inflammatory reactions, namely IL-1, TNF-a, IL-6, IL-11, IL-8 and other chemokines, G-CSF and GM-CSF. The cytokines known to mediate chronic inflammatory processes in turn can be divided into those participating in humoral inflammation (IL-3, IL-4, IL-5, IL-6, IL-7, IL-9, IL-10, IL-13 and transforming growth factor-β, TGF-β), and those contributing to cellular inflammation (IL-1, IL-2, IL-3, IL-4, IL-7, IL-9, IL-10, IL-12, interferons, IFNs, IFN-γ inducing factor also known as IGIF, TGF-β and TNF-α and -β). Moreover, while some cytokines act to worsen the disease by promoting systemic inflammation (pro-inflammatory cytokines), others serve to reduce inflammation and promote healing (anti-inflammatory cytokines). The pro-inflammatory cytokines reduce their biological activities (anticytokine therapy) by using neutralizing antibodies, soluble receptors, receptor antagonist and inhibitors of proteases that convert inactive precursors to active. The anti-inflammatory cytokines are a series of immunoregulatory molecules that control the pro-inflammatory cytokine response and play a physiologic role in inflammation and pathologic role in systemic inflammatory states.

Cytokine- and chemokine-mediated inflammation is a common denominator in neurodegenerative diseases. Neuroinflammatory processes significantly affect the nervous system by regulating the development, maintenance and sustenance of brain cells and their connections. Cytokines and chemokines are involved in the regulation of interactions between the central nervous system (CNS) and the immune system, and are important for the coordination of immune responses throughout the body. In the nervous system, cytokines and chemokines function as neuromodulators and regulate neurodevelopment, neuroinflammation and synaptic transmission. They are considered crucial for the brain’s immune function, as they serve to maintain immune surveillance, facilitate leukocyte trafficking and recruit other inflammatory factors. When stimulated by pathogens or abnormal cells, immune cells, as well as cells of the nervous system, can release cytokines and chemokines and respond to them via cytokine and chemokine receptors. While in the steady state, microglia (the resident macrophages of the brain) protect the nervous system by acting as scavengers for debris and microbial pathogens and by regulating innate and adaptive immune responses, in the pathological states within the nervous system (injuries, ischemic strokes and infections), the microglia are activated acting as a mediator of injury and death of neuronal and glial cells by producing pro-inflammatory factors such as cytokines and chemokines [4].

Considering that targeting the correct timing of an immune response is a pivotal factor in designing successful therapies, cytokines and chemokines have received considerable attention as therapeutic targets. Indeed, since cytokines and chemokines are vital for the normal functioning of the body, it is a challenge to understand the factors that dictate the switch from a protective to a deleterious inflammatory response. This helps to limit tissue damage and to design therapeutic agents to be delivered at a right dose in a timely manner that safely and effectively target only the detrimental mechanisms that contribute to disease pathogenesis [1,4].

Regarding the relevance of cytokines in cancer, pro-inflammatory counterworks are important at different stages of tumor development, particularly during invasion and metastasis. Immune cells and their signal molecules can influence all stages of tumor progression, as well as therapeutic intervention [12]. Inflammation is one of the mechanisms involved in creating pro-tumoral microenvironments in many different organs. While in normal tissue remodeling, chronic inflammation is terminated when repair is finished, in the tissues with carcinogenic mutations, compartments surrounding the epithelial component of tumor, cancer-associated fibroblasts, immune and inflammatory cells as well the vascular network and lymphatic spaces interact to create pro-inflammatory microenvironments, which are important drivers of all tumors. Although the exact mechanisms are still not entirely clear, it is known that TNF-α, IL-1α, IL-1β, IL-6, IL-10, IL-12, IL-17 and IL-23 act as important messengers in this process [12]. These cytokines have an important role in regulating inflammatory cells, especially macrophages, to create pro-tumoral microenvironments during chronic inflammation and provide the tumor with the ability to evade host responses. TNF-α, IL-6 and IL-17 have known roles in tumor growth and promotion; IL-6 and IL-10 are produced by tumor-associated macrophages and create conditions of immune suppression and angiogenesis; IL-1β and IL-6 promote angiogenesis and tumor invasion. Conversely, IL-2 is well-known to have antitumor activity via modulation of immune responses [12].

The pro- and anti-inflammatory cytokine (TNF-α and ILs) levels are also considered clinically relevant for the detection of heart failure (HF) and organ biocompatibility in patients with implanted left ventricle-assisted devices, as well as for the follow-up of chronic HF patients, where the higher circulating levels correlate with the severity of the disease [13,14].

## 3. Conventional Methods for the Determination of Cytokines

Currently, different analytical methods, including bioassays, immunoassays, molecular biology techniques and flow cytometry, are available for the determination of cytokines. Immunoassays, including the enzyme-linked immunosorbent assay (ELISA), radioimmunoassay, fluorescence immunoassay and time resolved immunofluorometric assay, have been widely employed for the detection and quantification of cytokines [9]. Among them, the “gold standard” methods are ELISA and bead-based immunoassay, whose signals are detected either by expensive flow cytometers or plate readers. Moreover, these methods require multiple steps, specialized personnel and assay times (sampling–detection) of 3–8 h. In addition, they are not compatible with decentralized or multiplexed determinations, features particularly demanded to advance in precision personalized medicine, also considering that the immune response may vary from individual to individual as well as the multifaceted roles of the target biomarkers [1,13,15].

Within this context, smart electrochemical biosensing is steadily gaining ground for the sensitive and accurate determination of these relevant biomarkers, while meeting the POCT requirements: simple but high-performance devices, economical in instrumentation and reagents, ability to provide single or multiplexed quantitative results with the required sensitivity and selectivity, simple interpretation by non-specialist users and with minimal actionable times at non-laboratory environments or with limited resources due to their low power consumption and ease of miniaturization [16,17].

Some review articles have been reported focusing on immunosensing [2] or label-free bioanalysis [1] using both optical and electrochemical detection. In addition, one paper reporting the use of electrochemical paper-based biosensors [18] for the determination of cytokines has been recently published. However, to our knowledge, there is not a comprehensive review on electrochemical biosensing strategies for the determination of cytokines involved in the inflammatory processes, underlying the diseases that threaten our society, including emerging infectious diseases such as the current global COVID-19 pandemic [19]. Therefore, through a representative sampling of the methods reported since 2015, this review article discusses in a comprehensive manner the state of the art and the potential and future directions of electrochemical biosensing for the analysis of cytokines. Photoelectrochemical (PEC)- or field-effect transistor (FET)-biosensing are not covered in this manuscript.

## 4. Bioelectroanalytical Methods for the Determination of Inflammation-Related Cytokines

Table 2 summarizes the analytical performance and the figures of merit of electrochemical biosensing strategies reported in the last five years (chronologically ordered) for the determination of cytokines, chemokines and receptors of relevance in the inflammation processes underlying autoimmune, neurodegenerative, cardiac, viral and cancer diseases as well as in hypercholesterolemia and diabetes.

As can be deduced from Table 2, several electroanalytical strategies have been developed based on the clever coupling of commercial bioreceptors (mainly antibodies and less frequently aptamers), attractive surface chemistries, nanomaterials and bioassay formats (direct and sandwich). These electroanalytical methods have been implemented both in magnetic beads or integrated formats and at both disposable and conventional electrodes. The electrochemical biosensing strategies make use of label-free or label-based configurations using different electrochemical techniques (mainly (chrono)amperometry, DPV, SWV and EIS and scarcely potentiometry) and electrode arrays or substrates (GCE, ITO, carbon, graphite and gold SPEs, Au microelectrodes and ISEs). As can be seen in Table 2, most of the label-free strategies, which are considered advantageous in terms of simplicity, cost and test time [1], have been developed in connection with impedimetric transduction [62,63].

The reported methods involve mainly the determination of TNF-α and IL-6, but also INF-γ, IL-8, IL-1β, IL-10, IL-13, IL-13Rα2, APN, RANKL and CXCL7. Targeted samples are mainly serum, but also saliva, which has distinctive advantages over serum in terms of non-invasive collection in any environment and by any user [14]. Electrochemical biosensing of cytokines and their receptors has also been carried out in blood, urine and cells or their secretions/supernatants and tissues.

It should be noted that despite the clinical relevance of the multiplexed determination of cytokines, there is only a limited number of works (about 1/5 of the methods summarized in Table 2) dealing with multiplexed strategies and just for the determination of two biomarkers.

In the last decade, electrochemical biosensing has resorted to different strategies to meet the increasingly demanding requirements of selectivity, sensitivity, simplicity, speed, portability, low assay cost and sample volume. Among these strategies, the use of magnetic particles and nanomaterials, and attractive surface chemistry to immobilize biomolecules and/or nanomaterials or to impart antifouling properties, is remarkable. The works summarized in Table 2 evidence these trends. In the following subsections, the relevant aspects of selected representative methods are discussed. Classification is made according to the use of specific materials (magnetic or non-magnetic) or chemistries, although one should be aware that some methods involve the use of more than one strategy to achieve better performance. A particular section is devoted to electrochemical biosensors exhibiting antibiofouling properties, a remarkable feature highly pursued nowadays.

### 4.1. Magnetic Particles-Assisted Electrochemical Biosensing for the Determination of Inflammatory Cytokines

Magnetic microparticles (MBs) have been widely used as solid supports in the construction of electrochemical biosensors, avoiding laborious protocols for the modification of electrodes, while providing significant improvements in assay time, sensitivity and minimization of sample matrix effect [64,65], as well as facilitating the quantification of biomarkers at the point-of-care (POC) and primary care settings [66]. Their nano-sized analogues (magnetic nanoparticles, MNPs) have been less used, because they are not as effective in avoiding loss during their modification and more prone to agglomeration [67,68].

Currently, screen-printed electrodes (SPEs) seem to be the preferred option to develop fast and cost-effective in vitro diagnosis methods with a large variety of applications [69], due to the fact they can be mass-produced from a variety of materials and in a customable way (different geometries, multiplexed formats, nanoparticulated and with suitable tailor-made functionalities) [70]. In addition, their small dimensions allow the use of small sample volumes and their flat shape facilitates the incorporation of magnetic bioconjugates on their surface in a stable and reproducible way by simple magnetic attraction.

As can be seen in Table 2, numerous electrochemical immunoassays have been developed for the single or simultaneous determination of cytokines combining the advantages of using MBs and SPEs [25,29,31,44,45,47,53,56].

Many of these strategies involve the formation of sandwich-type immune complexes on HOOC-MBs, enzyme amplification with HRP or AP and amperometric or DPV transduction. The methods were used for the single or dual determination of cytokines (TNF-, IL-8, TGF-1) and related receptors (IL-13Rα2) and achieved limit of detection (LOD) values in the range of pg mL^−1^–ng mL^−1^. In addition, they were applied to the analysis of human biofluids (commercial and real serum, urine and saliva, both spiked and non-spiked), colorectal cancer (CRC) cells (both lysed and intact) and paraffin-embedded tissues from CRC patients. Remarkable features of these methods are: (i) the use of an affibody as a capture bioreceptor [29]; (ii) the platform developed for the simultaneous determination of IL-8 and its associated mRNA in undiluted human saliva [31], which showed the potential of electrochemical platforms to perform multiomic determinations (in this particular case dual determination of a proteomic and a transcriptomic biomarker); (iii) the immunoplatform developed to improve the reliability of metastasis diagnosis through the simultaneous determination of the soluble and extracellular fraction of two proteins with opposite roles in cancer (the oncogenic IL-13Rα2 and the tumor suppressor E-CDH) [56]. This latter bioplatform met the required levels of sensitivity, selectivity and reproducibility for the determination of the target biomarkers both in liquid and solid biopsies and using a simple and shorter protocol (1 h 15 min) with 20–40 times smaller sample amount than the procedures involved in conventional ELISA methods. The developed method was applied to the simultaneous determination of the different fractions of both target proteins in a single run in serum (5 μL/determination) and tissues (0.5 μg/determination) from patients diagnosed with advanced CRC. The reported results showed the clinical potential of these biomarkers and their presence both on the surface of the tumor cells and in circulation. Importantly, the combined analysis of solid and liquid biopsies provides the most comprehensive and therapeutically valuable characterization of the tumor heterogeneity, which is crucial in diagnosis and prognosis of metastatic disease patients.

**Figure 1:**
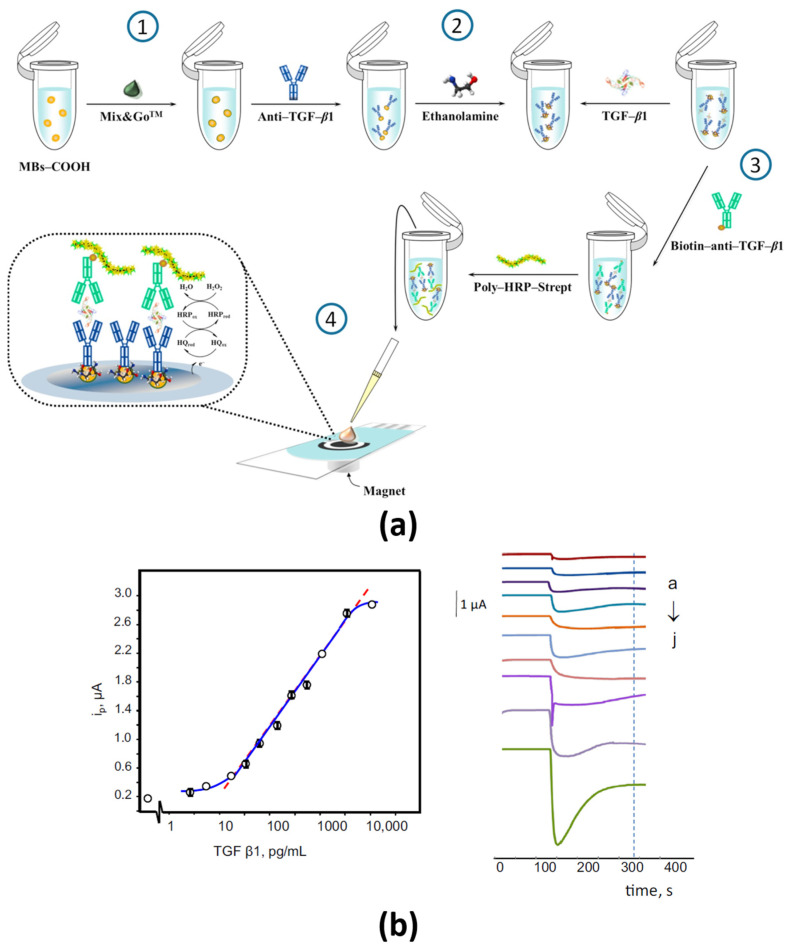
(**a**) Sandwich immunoassay involving covalent and targeted immobilization of CAb using the commercial Mix&Go polymer (a commercial polymeric coating that contains several metallic complexes able to bind proteins very efficiently) on HOOC-MBs, the use of an enzyme-conjugated biotinylated detection antibody with a Strep-HRP conjugate and amperometric transduction at SPCEs using the HRP/H_2_O_2_/HQ system and (**b**) calibration plot and real amperograms for TGF-β1 standards (a–j: 0–3000 pg mL^−1^). Reproduced from [44] with permission.

Figure 1 displays a scheme of the MBs-based immunoassay developed in 2017 for the determination of TGF-β1 [44]. This MBs-based immunosensor, which demonstrated applicability for the determination of TGF-β1 in spiked human urine, has been applied this year to the determination in the supernatants collected from top and bottom chambers of semipermeable transwells containing pericytes (PCs) and CRC cells (HCT116) [45]. The achieved results, which cannot be obtained with conventional ELISA methodology due to insufficient sensitivity, demonstrated that PCs played a key role in triggering the migration and invasion of CRC cells.

An interesting strategy makes use of a semi-automated, microfluidic immunoassay involving Strep-MBs coated with Btn-DAb and Strep-HRP and an 8-sensor array coated with glutathione (GSH)-AuNP for the multi-determination of TNF-α, IL-6, IL-1β and CRP in human serum samples from head and neck cancer patients [25]. Another illustrative example is the direct immunoassay with impedimetric transduction developed to determine IL-6 using commercial MBs modified with the bacterial antibody binding protein G (ProtG) on which the CAb was immobilized in an oriented manner across its Fc region [53].

**Figure 2:**
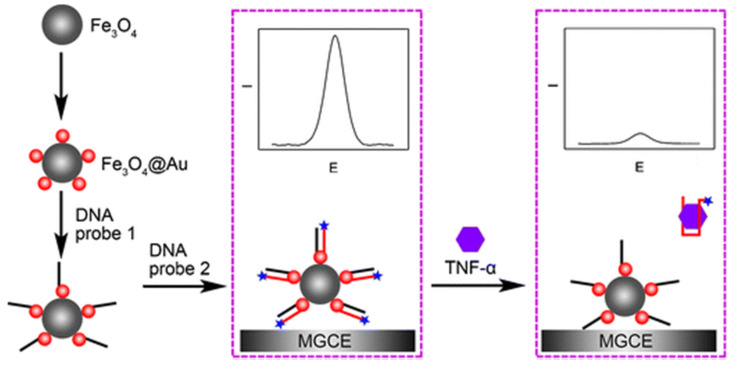
Fe_3_O_4_@AuNP assisted aptamer-based electrochemical method for the determination of TNF-α. Reproduced from [38] with permission.

Although less frequently, MNPs-based electrochemical biosensing strategies have also been proposed for the determination of cytokines [38,51]. Miao et al. [38] reported a biosensor based on the use of a specific aptamer and MNPs coated with AuNPs (Fe_3_O_4_@AuNPs) for the determination of TNF-α. As is shown in Figure 2, Fe_3_O_4_@AuNPs were modified with a thiolated DNA probe complementary to an aptameric sequence labeled with methylene blue (MB) which is released in the presence of the target analyte (TNF-α), thus leading to a significant decrease of the MB oxidation signal monitored by SWV upon Fe_3_O_4_@AuNPs deposition on a magnetized glassy carbon electrode (MGCE). The Fe_3_O_4_@AuNPs assisted aptamer-based method achieved an LOD of 10 pg mL^−1^ and was employed in the analysis of spiked human serum samples. Moreover, a sandwich immunoassay involving HRP-DAb and MNPs coated with poly(pyrrole-co-pyrrole-2-carboxylic acid) electrodeposited onto an 8 × Au-SPEs array was proposed for the chronoamperometric determination of TNF-α using the system HRP/H_2_O_2_/TMB with an LOD of 0.3 pg mL^−1^ [51].

### 4.2. Integrated Biosensors Involving Surface Chemistries for the Determination of Inflammatory Cytokines

Integrated bio-scaffolds also provide an interesting route for developing electrochemical biosensors. Their analytical performance can be significantly improved by applying rational surface chemistries and/or by coupling with smart nanomaterials used as both electrode modifiers or advanced labels. In this context, the use of the diazonium salt chemistry allows the development of simple, rapid and versatile methods imparting interesting features to the modified substrates such as antibiofouling [71]. This strategy of modification is a powerful tool to immobilize in a stable and reproducible way a wide range of biomolecules or nanomaterials useful to modify a wide variety of electrode surfaces with different substituents. In addition, due to the electrografting processes, it allows differential functionalization of closely spaced surfaces to construct multianalyte biosensors [72]. Furthermore, the copper(I)-catalyzed azide-alkyne cycloaddition (CuAAC) has a great variety of possibilities in electrochemical biosensors [73]. To overcome the long time needed for chemical generation of the cuprous ion (~16 h), the electrogeneration of the catalyst by applying a previously optimized reduction potential for a fixed time (~300 s) has been exploited, leading to a methodology known as electroclick chemistry that provides good results in the preparation of biosensors [59,74].

Table 2 shows that both diazonium salts-based electrografting and click and electroclick chemistries have been used for the construction of electrochemical biosensors for inflammatory cytokines. These methods involved the functionalization of carbon nanomaterials [27,41,42,43,52] and electrode surfaces [11,13,14,26,36,43,46,52,54,55,57,58,59,60,61] or the incorporation of ethynyl-functionalized antibodies [41,57,59].

The use of these chemistries for the functionalization of electrode substrates include SPEs (carbon or gold) modified with *p*-aminobenzoic acid (*p*-ABA) [26,43,52,54,55,57,58,60,61] and 4-carboxymethyl aryl diazonium (CMA)-modified Au microelectrodes [13,14,32,46,51]. The grafted electrodes have been used mainly for covalent immobilization of the capture bioreceptor and occasionally for immobilization of nanomaterials [52].

**Figure 3:**
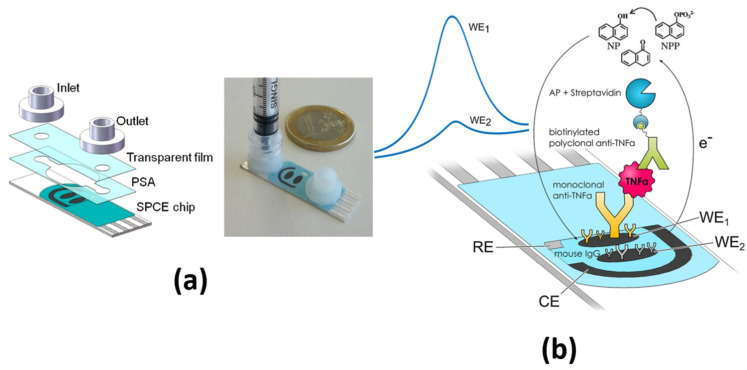
(**a**) Picture and real image of an electrochemical biochip and (**b**) schematic display of the developed sandwich immunoassay for the determination of TNF-α. Reproduced from **[26]** with permission.

An interesting example is the simple, rapid and disposable electrochemical microfluidic immuno-biochip developed by Eletxiguerra et al. for the determination of TNF-α [26]. For this purpose, *p*-ABA-grafted SPdCEs were biofunctionalized with appropriate antibodies and subsequently encapsulated with an all-disposable polymeric microfluidic cell (Figure 3a). One of the working electrodes was functionalized with a specific antibody for the target cytokine and the other with a non-specific antibody. This latter was used as a negative control to serve as baseline, thus allowing the direct TNF-α determination through a sandwich immunoassay involving a Btn-DAb and Strep-AP in a single measurement with no need for calibration curves (Figure 3b). Using DPV in the presence of 1-NP, this immuno-biochip provided an LOD of 4.1 ng mL^−1^ and was applied to the analysis of four-times diluted human serum samples.

**Figure 4:**
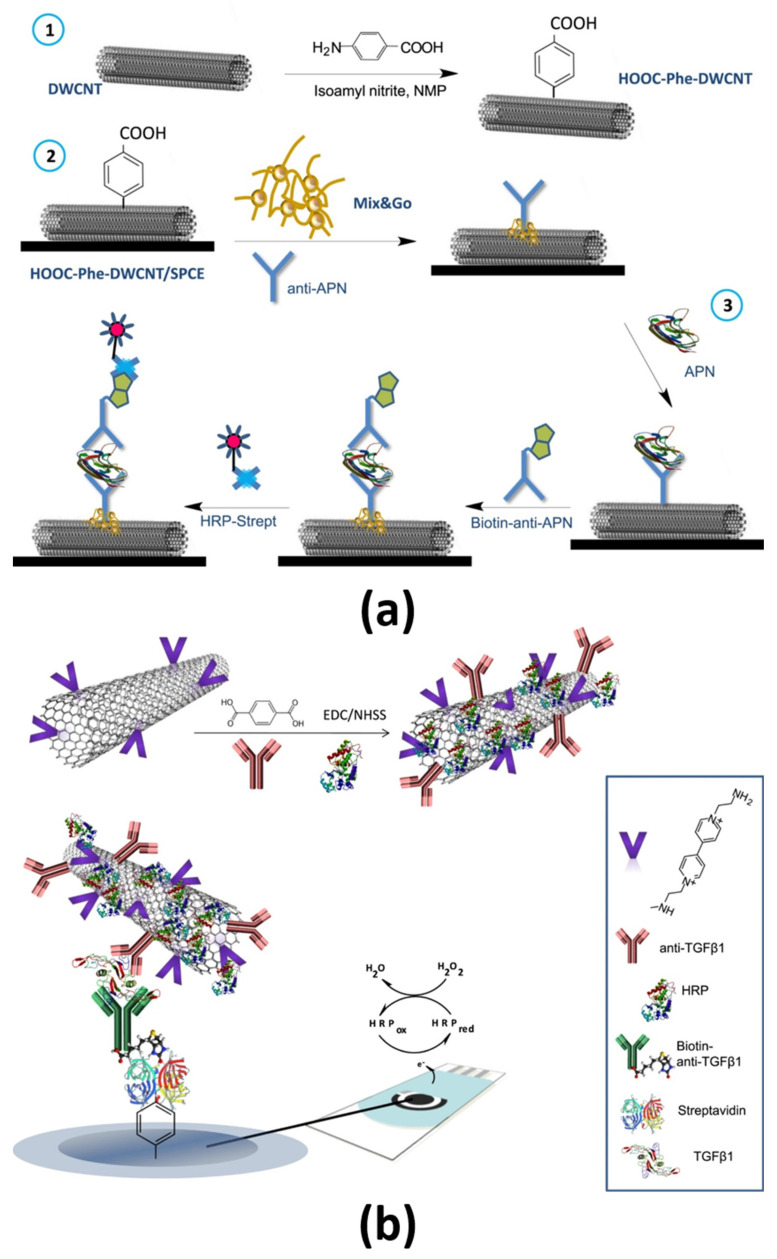
(**a**) Modification of SPCE with *p*-ABA-DWCNTs and (**b**) use of V-Phe-SWCNTs as nanocarriers of DAb and HRP molecules for the preparation of amperometric sandwich immunoplatforms for the determination of APN and TGF-β1, respectively. Reproduced from (**a**) [27] and (**b**) [43] with permission.

**Figure 5:**
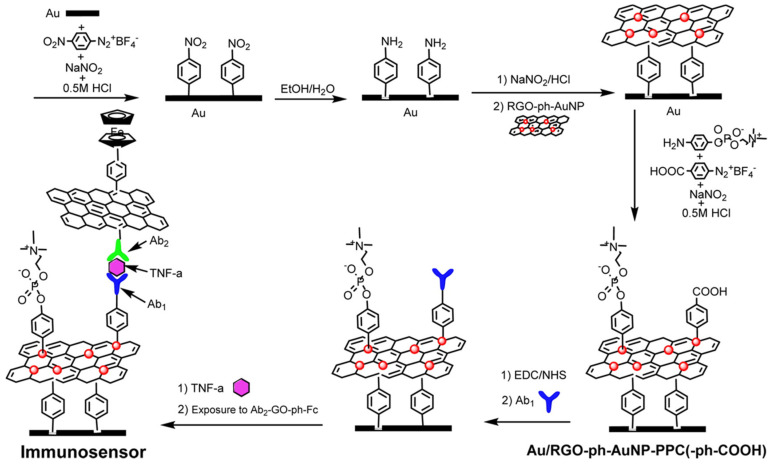
Sandwich-based immunosensor for the determination of TNF-α involving the use of Au-RGO-ph-AuNP-ph-PPC(-ph-COOH) as electrode modifiers and DAb-GO-ph-Fc as tracers. Reproduced from [34] with permission.

Aryl-diazonium chemistry has been exploited to functionalize carbon nanotubes (CNTs) [27,42,43] and reduced graphene oxide (RGO) [34]. In fact, *p*-ABA-DWCNTs were used as SPCE modifiers (Figure 4a) [27,42] and V-Phe-SWCNTs as nanocarriers (Figure 4b) [43] to construct electrochemical immunoplatforms for the single determination of APN or TGF-β1 and the dual determination of IL-1β + TNF-α. Qi et al. [34] proposed an interesting immunosensor for the determination of TNF-α using as the sensing interface Au substrates modified by covalent assembly with AuNP-loaded RGO nanocomposites prepared by aryldiazonium salt chemistry (RGO-ph-AuNP). This strategy allowed loading a large amount of CAb and antifouling 4-aminophenyl phosphorylcholine (PPC) molecules (Au/RGO-ph-AuNP-PPC(-ph-COOH)/CAb) as well as the use of GO nanocomposites modified with DAb and 4-ferrocenylaniline (DAb-GO-ph-Fc) as tracers (Figure 5).

**Figure 6:**
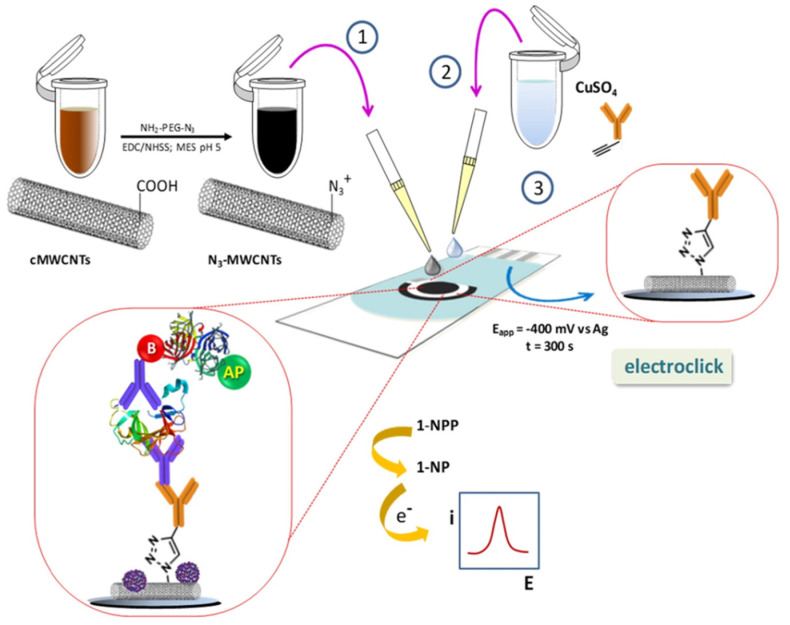
Sandwich immunoplatform for the DPV determination of IL-1β using IgGs-MWCNTs prepared by electro-click chemistry as SPCE modifiers. Reproduced from [59] with permission.

Moreover, immunoscaffolds reported for the determination of inflammatory cytokines and chemokines involved in click [42,57] or electro-click (Figure 6) [59] chemistry to prepare IgGs-MWCNTs by reaction of azide-functionalized MWCNTs and ethynyl-IgGs.

The electrochemical immunoplatforms prepared using electrografting and click or electro-click chemistry exhibited analytical characteristics compatible with clinical applications providing LODs in the low pg mL^−1^–ng mL^−1^ level, and were employed to perform the analysis of the target cytokines/receptors (TNF-α, APN, TGF-β1, IL-6, IL-13Rα2, IFN-γ, RANKL, IL-1β and IL-10) in different human biofluids (saliva, serum, plasma and blood).

### 4.3. Electrochemical Biosensing Methods Involving Nanomaterials for the Determination of Inflammatory Cytokines

Regarding nanomaterials, their multifunctional nature facilitates the improvement of several key features in electrochemical bioassays, including sample treatment, analyte capture, signal amplification and transduction. They have been widely used as electrode modifiers to improve the immobilization of bioreceptors and the charge transfer as well as advanced labels able to carry large amounts of electroactive reporters to amplify the electrochemical signals [75].

The nanostructures used in electrochemical biosensing strategies reported in the last five years for the determination of cytokines include metallic (AuNPs, Ag@Pt), metal oxide (TiO_2_), magnetic and polymer (PPyNP) nanoparticles and carbon nanomaterials (CNTs, rGOs, GQDs, C_60_) either as single nanostructures or combined in hybrid nanostructures (Fe_3_O_4_@AuNPs, AuNPs/MWCNTs, GQDs/MWCNTs) as well as with other modifiers such as chitosan (CS), carboxymethylcellulose (CMC) and ionic liquids (ILs) displaying synergic properties.

**Figure 7:**
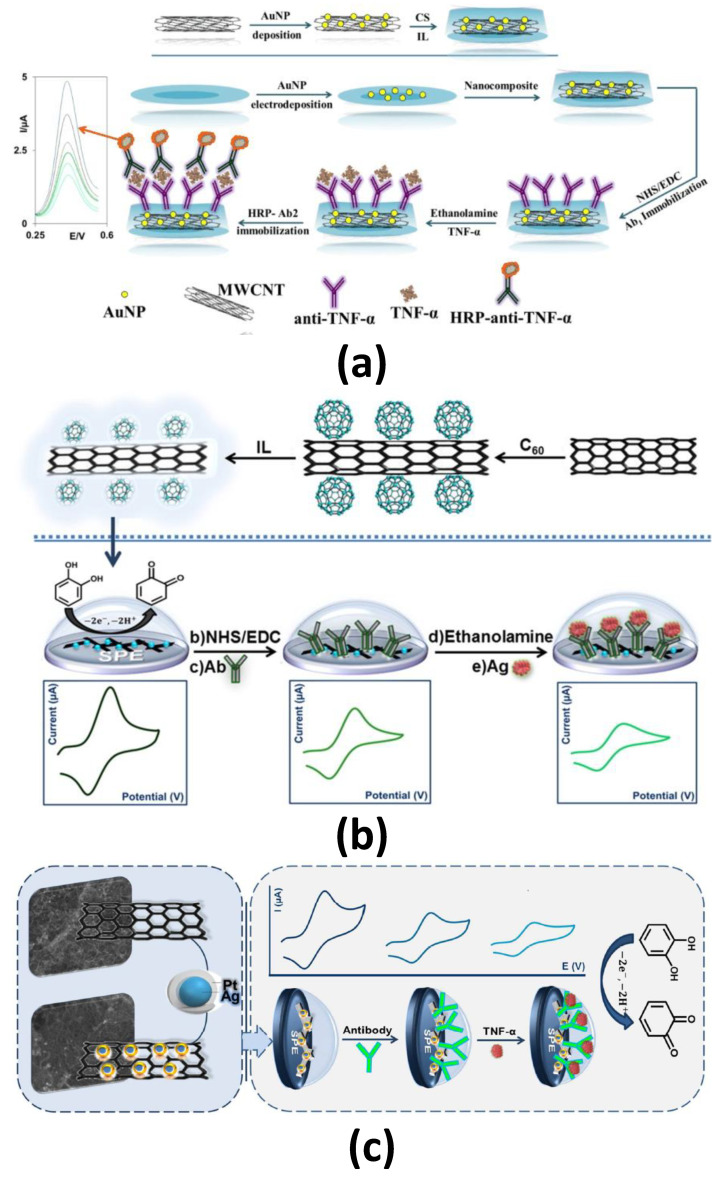
Integrated immunosensors for the determination of TNF-α using (**a**) AuNP/MWCNT-AuNP nanocomposite/IL-CS composite film, (**b**) Ag@Pt-CNTs-CS and (**c**) C_60_-CNTs-IL nanocomposites as electrode modifiers. Reprinted from (**a**) [20] (**b**) [21] and (**c**) [22].

Regarding the use of nanomaterials as electrode modifiers, illustrative examples are the immunosensors developed by Mazloum-Ardakani et al. for the determination of TNF-α [20,21,22] by exploiting the advantages of AuNP/MWCNT-AuNP nanocomposite/IL-CS composite film, Ag@Pt-CNTs-CS and C_60_-CNTs-IL nanocomposites as modifiers of GCE and GSPE and using sandwich or direct formats and DPV detection in the presence of AP/H_2_O_2_/acetaminophen (Figure 7a) or catechol (Figure 7b,c), respectively. All these immunosensors provided LOD values of ~2.0 pg mL^−1^ and were applied to the determination of TNF-α in spiked human serum samples.

Arenas et al. [30] reported a sandwich-type immunosensor for the determination of APN by immobilizing the CAb at CMC–rGO/SPCEs in a stable and oriented way through the metal complexes-based polymer Mix&Go^TM^. In addition, Btn-DAb + Strep-HRP and amperometry in the presence of H_2_O_2_/HQ were used, achieving an LOD of 61 ng mL^−1^. The immunosensor was applied to the analysis of human serum from hypercholesterolemia and diabetes patients. Other attractive electrochemical affinity biosensors have been reported by utilizing AuNPs-AuE [33], AuNP-rGO-ITO [35], AuNPs/PPyNPs/SPGE [37] and SWCNT-WE [49]. These affinity biosensors include aptasensors for IL-6 [33,37] and immunosensors for TNF-α [35], involving direct formats and impedimetric transduction, as well as an amperometric sandwich immunosensor design for IL-13 [49].

Regarding the use of nanomaterials as nanocarriers, MWCNTs-based nano-hybrids stand out. For instance, GQDs/MWCNTs hybrids, formed by the strong π–π interactions of MWCNTs and GQDs, merge the excellent properties of the individual nanomaterials in terms of remarkable conductivity, biocompatibility, high density of active groups for the immobilization of biomolecules and intrinsic peroxidase-like activity. These nanohybrids have been applied as nanocarriers of signaling elements (DAb and HRP molecules) in integrated immunoplatforms prepared at p-ABA grafted-SPCEs for the determination of IL-13Rα2 [54,55]. An eight-times improved sensitivity was achieved by using a nanohybrid composed of MWCNTs decorated with AuNPs as a similar nanocarrier for the development of an amperometric immunosensor for the determination of RANKL [60]. Indeed, amplification factors of 6.5 and 29.7 are observed when MWCNTs and AuNPs/MWCNTs were used as nanocarriers of signaling elements in a sandwich immunosensor for the amperometric determination of RANKL in comparison with the conventional enzymatic labeling of the DAb with an HRP-secondary antibody (unpublished results). These results are in good agreement with those reported by Rusling’s group, which declared a ~5-fold increase in sensitivity by forming sandwich immunocomplexes using MWCNTs decorated with HRP and DAb in comparison with the common Btn-DAb-Strept-HRP approach [76].

**Figure 8:**
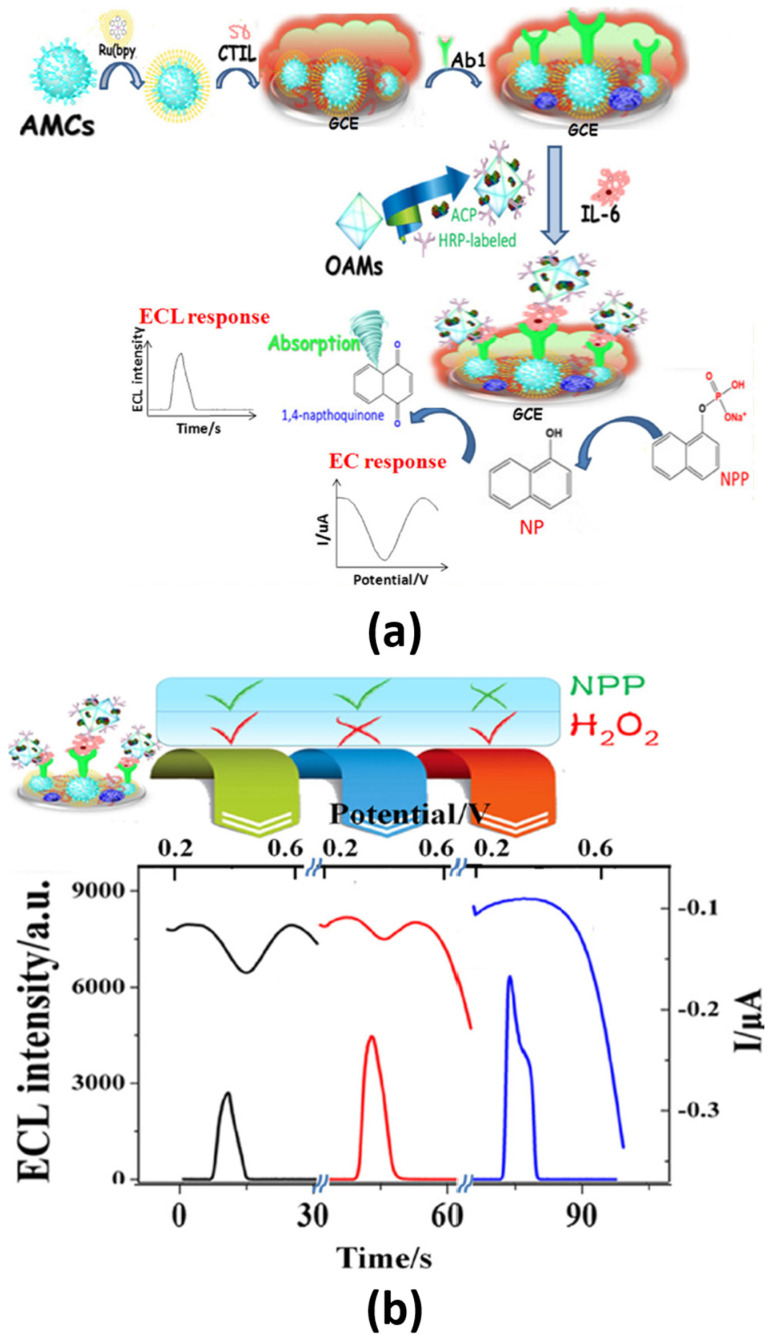
(**a**) Electrochemical and ECL dual response sandwich immunosensor involving different TiO_2_ nanoarchitectures both as electrode modifiers and nanocarriers; (**b**) real DPV and ECL-time curves obtained with the developed immunosensor in buffer containing 5 mM NPP and 15 mM H_2_O_2_ (black curves), 5 mM NPP only (red curves) and 15 mM H_2_O_2_ only (blue curves). Reproduced from [48] with permission.

Some immunosensors combine the use of nanomaterials as electron modifiers and as signaling element nanocarriers in the same device. For example, Liu et al. reported a dual-responsive (combining ECL and electrochemical detection) sandwich-type immunosensor for the determination of IL-6 using two kinds of TiO_2_ mesocrystal nanoarchitectures: a composite prepared from TiO2 (anatase) mesocages (AMCs) and a carboxy-terminated ionic liquid (CTIL) as a modifier of a GCE, and octahedral anatase TiO_2_ mesocrystals (OAMs) as a matrix for immobilizing acid phosphatase (ACP) and secondary antibody (Ab_2_) labeled with horseradish peroxidase (HRP) (Figure 8) [48]. The oxidation of 1-NP, produced in situ on the surface of the GCE due to the hydrolysis of added 1-NPP by ACP, by HRP in the presence of H_2_O_2_ was monitored by DPV providing an LOD of 0.32 fg mL^−1^. Unfortunately, the immunosensor was not yet applied.

### 4.4. Electrochemical Biosensors with Antibiofouling Properties for the Determination of Inflammatory Cytokines

**Figure 9:**
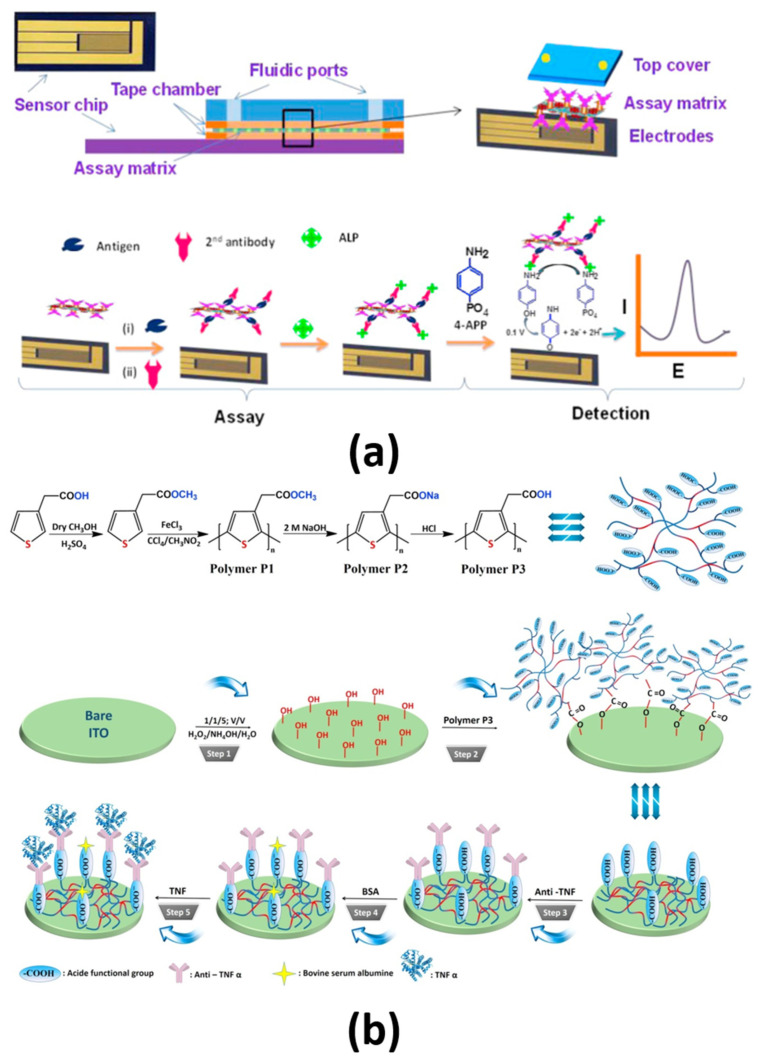
(**a**) Schematic display of the on-chip electrochemical immunoassay platform developed for the determination of TNF-α in undiluted serum using off-surface membrane matrix (upper row) and DPV detection in the presence of 4-APP (lower row). (**b**) Antibiofouling impedimetric immunosensor for the determination of TNF-α constructed with a semi-conductive and carboxylated polymer-modified ITO thin film: polymer preparation (upper row) and immunosensor preparation and recognition (lower rows). Reproduced and adapted from (**a**) [36] and (**b**) [39] with permission.

Beyond sensitivity and selectivity, nowadays, antifouling is a pursued characteristic to allow the adequate functioning of electrochemical biosensors in real world matrices without compromising their performance and involving simple and straightforward protocols [71,77]. Most common recent strategies in the development of electrochemical sensors exhibiting antifouling properties include surface functionalization with molecular systems through self-assembly, electrografting and polymerization [71]. As can be seen in Table 2, electrochemical biosensors prepared for the determination of cytokines with proven antibiofouling properties involve the use of mixed layers of zwitterionic aryl diazonium salts derivatives [28], a 2D PC membrane as an off-surface matrix [36] and (semi)conducting polymers [15,39]. An ITO electrode was modified with mixed layers of zwitterionic phenyl phophorylcholine to repel nonspecific protein adsorption, and phenyl butyric acid to immobilize the capture antibody. This bioscaffold was employed to develop a sandwich immunosensor to determine TNF-α by chronoamperometry using the HRP/H_2_O_2_/ferrocenemethanol system, in undiluted whole blood at the low pg mL^−1^ level with results in good agreement with those obtained using a commercial ELISA kit [71]. Arya et al. [36] reported an on-chip electrochemical immunoassay platform for the determination of TNF-α prepared with a porous polycarbonate (PC) two-dimensional (2D) membrane-based off-surface matrix (Figure 9a). The 2D PC, with the CAb covalently attached using 4-fluoro-3-nitro-azidobenzene (FNAB) as a cross-linker, was integrated over an array of micro fingers of a gold sensor chip and with a fluidic system for reagent flow and incubation chamber. Using a sandwich format involving Btn-DAb and Strep-AP and DPV detection in the presence of 4-APP, the immunoplatform allowed the determination of the target cytokine in the pg mL^−1^ range in undiluted serum. A highly sensitive impedimetric immunosensors was developed by Sezgintürk’s research group using ITO thin films coated with conducting polymers (poly(3-thiophene acetic acid), P3 in Figure 9b [39] or poly(pyrrole N-hydroxy succinimide) (PPyr-NHS) [15]) to act as immobilization matrices. The immunosensor exhibited rapid and sensitive determination (LODs in the low fg mL^−1^) of the target cytokines (TNF-α [39] and IL-6 [15]) and were used for the analysis of the cytokines in human saliva and serum samples.

### 4.5. Other Electrochemical Biosensing Methods for the Determination of Inflammatory Cytokines

There are also other electrochemical biosensing strategies, without using magnetic particles, nanomaterials or surface chemistries, suitable for the determination cytokines. For example, Say et al. [23] reported a potentiometic sensor for TNF-α based on the use of a PVC membrane with ruthenium-based antibodies nanoparticles (prepared by the microemulsion polymerization technique and cross-linking the specific antibodies with ruthenium chelating agents) dispersed in dibutyl phthalate (DBP) embedded tissues. Through the measurement in the potential changes of the Ru(III)/Ru(II) pair after target protein recognition, the sensor provided an LOD of 0.015 mg L^−1^ and was applied to the analysis of RA patients’ plasma samples.

**Figure 10:**
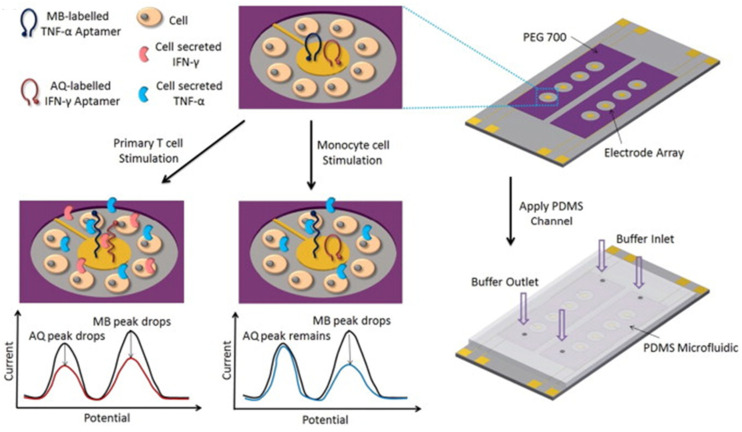
Schematic display of the microfluidic sensing platform using aptameric sensors for the dynamic monitoring of IFN-α and TNF-α from immune cells. Reproduced from [24] with permission.

An electrochemical aptasensor for the dual determination of IFN-γ and TNF-α was prepared by immobilizing specific hairpin aptamers dually labeled with thiol and redox reporters (anthraquinone (AQ) for IFN-γ or methylene blue (MB) for TNF-α) on gold electrodes integrated into microfluidic devices to dynamically monitor cytokine release from cells (Figure 10) [24]. Binding of the target cytokine caused a conformational change in the aptamer, which resulted in a decrease of the redox current measured by SWV of the redox reporter.

Simple immunosensors with good analytical performance have been reported for the determination of TNF-α and IL-6 using sandwich and direct formats by covalent immobilization of capture antibodies on SAM-modified Au microarrays of thiolated cross-linkers able to bind primary amines through formation of amide bonds ((3, 3′-dithiodipropionic acid di(N-hydroxysuccinimide ester) [40] and sulfosuccinimidyl 6-(3’-[2-pyridyldithio]-propionamide)hexanoate [50]). Using DPV and EIS detection, these immunoplatforms provided LOD values of 60 pg mL^−1^ (TNF-α) and 0.95 pM (IL-6) and were applied to the determination in spiked human serum samples.

## 5. General Considerations, Challenges and Outlook

Bearing in mind that cytokines are involved in several of the diseases of concern to our society nowadays, the interest in their determination and in the implementation of competitive methodologies for this purpose will not wane. As already commented, it is essential to perform their multiplexed determination with tools able to satisfy the demands of the current clinic in terms of the simplicity of use, affordable cost and suitability for decentralized analysis by any user.

Electrochemical biosensing technologies are evolving and maturing in amazing ways to meet many of these demands. Indeed, cytokines, as target biomarkers, are in vogue in electrochemical biosensing applications, as is evident by the upsurge in the number of research articles devoted to the determination of cytokines in the past few years.

The incorporation of materials, other types of modifiers, chemicals and attractive bioassay formats into their design has played a decisive role in the opportunities and capabilities provided by electrochemical biosensing. In order to give a realistic picture of the state of the art, this review article discusses the significant advances and features achieved in the field through selected representative methods reported in the last five years. These methods show that both magnetic particles-based and integrated electrochemical bioplatforms exhibit compatible characteristics in terms of sensitivity and selectivity with the determination of these biomarkers in the clinic.

The use of magnetic particles, particularly those of micrometric size, as solid supports for the implementation of bioassay configurations, combined with electrochemical transduction on SPEs has shown the suitability to perform multiomic determinations (simultaneous determination of IL-8 and its associated mRNA) and to analyze cytokines in small amounts of complex biological samples (raw serum, whole cells and tissue extracts), in clinically actionable times and using simple test protocols.

In the case of integrated formats, coupling with nanomaterials and a rational modification of electrode surfaces with suitable chemicals and modifiers play particular relevant roles. Electrografting of diazonium salts, click and electroclick chemistries provide a great versatility and ability for the functionalization of carbon nanomaterials and electrode surfaces, and in the incorporation of ethynyl-functionalized antibodies. The electrode functionalization, together with the use of SAMs of zwitterionic compounds, PC membrane-based off-matrix and (semi-)conducting polymers, makes it possible to impart anti(bio)fouling properties to biosensors, which is highly demanded to ensure their adequate performance in real world matrices. A variety of nanomaterials, either used singly, or combined in hybrid nanostructures or with other modifiers (CS, CMC and ILs), have been employed as electrode modifiers to improve bioreceptor immobilization and charge transfer, as well as advanced labels to carry large numbers of electroactive reporters.

The reported bioelectroanalytical strategies exhibit high sensitivity (usually in the order of ng mL^−1^, but in some cases also fg mL^−1^) and some of them have been successfully integrated into microfluidic devices. In addition, the methods have been applied to the determination of cytokines in un-pretreated whole blood and saliva, whole cells, cell lysates or secretions and paraffin tissue extracts.

Despite the current demand for multiplexed determinations to improve the reliability of diagnosis and clinical prognosis, which is particularly relevant in this family of compounds, it should be noted that only one in five of the reported methods addressed the challenge to simultaneously screen cytokines (and two or three at most). Furthermore, the application to real samples is still limited or has been addressed with a small number of samples and only at research level. Future efforts in this area should be carried out in close coordination with different actors in the clinic and society, and focused on exhaustive validations of both candidate biomarkers (existing and others that can be identified) and developed devices. This combined effort will be essential for the future implementation of these strategies in the market, clinic and daily life. At the same time as working on the validation of the biomarkers and the corresponding devices, one must not forget to continue advancing in the development of other strategies exploiting the use of other bioreceptors such as biomolecular switches and nanomaterials with mimicked enzyme activity (nanozymes). This could open the way for reagentless biodevices, with the capacity for almost real time determinations and of lower cost and more robust to variations in pH and temperature than those involving natural enzymes.

Despite the many challenges lying ahead, we are pretty convinced that the continuous advancements in electrochemical biosensing technology will provide user-friendly but powerful tools suitable for adoption in real clinical settings, at which point screening programs and personalized medicine will become a reality, to assist in the diagnosis, prognosis and monitoring of diseases facing our society, by determining the cytokines involved in the autoimmune and inflammatory processes occurring during their starting and evolution.

## Figures and Tables

**Table 1:** Main functions and examples of cytokines.

Family	Functions	Examples
Cytokines	Pro-inflammatory; ↑ inflammatory mediators; ↑ innate immune responses	IL-1α, IL-1β, TNF-α, IL-12, IL-18, IL-23
	Anti-inflammatory; ↓ inflammatory genes; ↓ cytokine-mediated lethality	IL-10, IL-13, TGF-β, IL-22, IL-1Ra, IFNα/β
	Angiogenic; neovascularization; pro-metastatic	VEGF, IL-1, IL-6, IL-8
	Osteoclast activation	RANKL
Chemokines	↑ Cellular emigration; ↑ cell activation	IL-8, MCP-1, MIP-1α, CC and CXC chemokines
Interferons (IFNs)	Type I: anti-viral immunity; ↑ class I MHC; anti-inflammatory; anti-angiogenic	IFNα, IFNβ, IFNω
	Type II: macrophage activation; increase class II MHC IFNγ also ↑ class I MHC and is responsible for anti-viral immunity (stimulates CD8^+^ T cells/Th1 responses).	IFNγ
Adipokines	Pro-inflammatory; ↓ autoimmune disease pro-atherogenic	IL-1α, TNF-α, IL-6, leptin, adiponectin, resistin
Tumor necrosis factors	Pro-inflammatory pyrogenic; non-specific immunity; apoptosis	TNF-α, TNF-β
Mesenchymal growth factors	Fibrosis; pro-metastatic	FGF, HGF, TGF-β, BMP
Colony stimulating factors	Hematopoiesis; pro and anti-inflammatory	IL-3, IL-7, G-CSF, GM-CSF, M-CSF
Nerve growth factors	↑ nerve/Schwann cells; B-cell activation	BNDF, NGF

MHC: major histocompatibility complex.

**Table 2:** Electrochemical biosensing methods reported since 2015 for the determination of inflammatory cytokines.

Electrode	Fundamentals	Detection Technique	Target Analyte/Disease	LR/LOD	Sample	Ref.
GCE	Sandwich-based immunosensor involving HRP-DAb at a GCE modified with an AuNP/MWCNT-AuNP nanocomposite/IL-CS composite film	DPV (H_2_O_2_ + acetaminophen)	TNF-α	6.0–100 pg mL^−1^/2.0 pg mL^−1^	Spiked human serum	[20]
GSPE	Direct immunosensing at a graphite SPE modified with Ag@Pt-CNTs-CS	DPV (catechol)	TNF-α	6.0–60 pg mL^−1^/1.6 pg mL^−1^	Spiked human serum	[21]
GSPE	Direct immunosensing at a graphite SPE prepared by entrapping the CAb onto a C_60_–CNTs–IL nanocomposite	DPV (catechol)	TNF-α	5.0–75 pg mL^−1^/2.0 pg mL^−1^	Spiked human serum	[22]
ISE	Potentiometic sensor based on ruthenium-based antibodies nanoparticles	Potentiometry (Ru(III)/Ru(II))	TNF-α/RA	0.1–1.0 mg L^−1^/0.015 mg L^−1^	Plasma	[23]
Electrodes integrated into microfluidic devices	Direct aptasensing using aptamers labeled with AQ and MB	SWV (AQ + MB)	INF-γ + TNF-α	INF-γ: 9–130 ng mL^−1^/6.35 ng mL^−1^ TNF-α: 9–88/5.46 ng mL^−1^	Dynamically monitoring of cytokine release from immune cells	[24]
Array of eight SPCEs	Semiautomated, microfluidic immunoarray involving the use of Strep-MBs coated with Btn-DAb and Btn-HRP and an 8-sensor array coated with GSH-AuNP	Amperometry (HRP/H_2_O_2_/HQ)	TNF-α, IL-6, IL-1β, CRP/Cancer	IL-6:	Human serum from head and neck cancer patients	[25]
				Up to 4.5 pg mL^−1^/18 fg mL^−1^		
				TNF-α:		
				Up to 12 pg mL^−1^/10 fg mL^−1^		
				CRP:		
				Up to 11 pg mL^−1^/15 fg mL^−1^		
				IL-1β:		
				Up to 22 pg mL^−1^/40 fg mL^−1^		
SPdCE encapsulated with an all-disposable polymeric microfluidic cell	Sandwich-based immunosensor involving Btn-DAb and Strep-AP developed at *p*-ABA grafted SPdCE	DPV (AP/1-NP)	TNF-α	13.7–50.0 ng mL^−1^/4.1 ng mL^−1^	Real human serum	[26]
SPCE	Sandwich-based immunosensor involving Btn-DAb and Strep-HRP at a SPCE modified with *p*-ABA-DWCNTs	Amperometry (HRP/H_2_O_2_/HQ)	APN	0.05–10.0 μg mL^−1^/14.5 ng mL^−1^	Human serum from healthy subjects	[27]
ITO	Sandwich-based immunosensor involving HRP-DAb at an ITO electrode modified with mixed layers of PPC and PBA	Chronoamperometry (HRP/H_2_O_2_/Ferrocenemethanol	TNF-α	0.01–500 ng mL^−1^/10 pg mL^−1^	Non-pretreated whole blood	[28]
SPCE	Sandwich type bioassay implemented on the surface of HOOC-MBs using and affibody as capture bioreceptor a DAb and an AP-anti-mouse IgG	DPV (AP/1-NPP)	TNF-α	--/0.038 ng mL^−1^	Spiked commercial human serum	[29]
SPCE	A sandwich-type immunoassay involving the commercial metal complexes-based polymer Mix&Go^TM^ for the stable and oriented immobilization of CAb at CMC–rGO/SPCEs and	Amperometry (HRP/H_2_O_2_/HQ)	APN	0.5–10.0 μg mL^−1^/61 ng mL^−1^	Human serum from hypercholesterolemia or diabetes patients	[30]
SPdCE	Direct hybridization assay using a Btn-hairpin DNA Cp and sandwich immunoassay involving Btn-DAb and Strep-HRP implemented on the surface of Strep-MBs and HOOC-MBs	Amperometry (HRP/H_2_O_2_/HQ)	IL-8 mRNA + IL-8 protein	IL-8 mRNA: 0.32–7.5 nM/0.10 nM IL-8: 87.9–5000 pg mL^−1^/26.4 pg mL^−1^	Raw human saliva	[31]
Eight Au working microelectrodes	Direct immunosensing at CMA-modified Au microelectrodes	EIS ([Fe(CN)_6_]^4−/3−^]	TNF-α/HF	1–15 pg mL^−1^/--	Artificial saliva	[32]
AuE	Direct aptasensing at a thiolated aptamer-modified AuNPs-AuE	EIS ([Fe(CN)_6_]^4−/3−^]	IL-6	0.02 pg mL^−1^–20 pg mL^−1^/--	Spiked artificial sweat	[33]
AuE	Sandwich-based immunosensor involving the use of Au-RGO-ph-AuNP-ph-PPC(-ph-COOH) as electrode modifiers and DAb-GO-ph-Fc as tracers	SWV (Fc)	TNF-α	0.1−150 pg mL^−1^/0.1 pg mL^−1^	Live BV-2 cells secretions	[34]
ITO microelectrode array	Direct immunosensing at ITO microelectrode array nanostructured with AuNP-rGO hybrids	EIS ([Fe(CN)_6_]^4−/3−^]	TNF-α	1–1000 pg mL^−1^/0.43 pg mL^−1^	--	[35]
Array of micro fingers of gold	Sandwich immunosensor involving Btn-DAb and Strep-AP and covalent attachment of CAb on 2D PC membrane based off-matrix achieved FNAB cross-linker	DPV (4-APP)	TNF-α	100 pg mL^−1^–100 ng mL^−1^/100 pg mL^−1^	Undiluted serum	[36]
SPGE	Direct aptasensing at a AuNPs/ PPyNPs/SPGE	EIS ([Fe(CN)_6_]^4−/3−^]	IL-6	1 pg mL^−1^–15 μg mL^−1^/0.33 pg mL^−1^	Spiked human serum	[37]
GCE	Direct aptasensing approach at Fe_3_O_4_@AuNP modified with an SH probe hybridized with a MB-labeled aptameric probe	SWV (MB)	TNF-α	10 pg mL^−1^–100 ng mL^−1^/10 pg mL^−1^	Spiked human serum	[38]
ITO	Direct immunosensing at a Poly(3-thiophene acetic acid)-modified ITO	EIS ([Fe(CN)_6_]^4−/3−^]	TNF-α	0.01 pg mL^−1^–2 pg mL^−1^/3.7 fg mL^−1^	Human saliva and serum	[39]
Comb-shaped gold electrode microarray	Sandwich-based immunosensor involving Btn-DAb and Strep-AP at a DTSP/Au electrodes	DPV (4-APP)	TNF-α	500 pg mL^−1^–100 ng mL^−1^/60 pg mL^−1^	Spiked undiluted serum	[40]
MWCNTs-SPCE	Sandwich-based immunosensor involving Btn-DAb and Strep-HRP and click chemistry-assisted cAb immobilization on IgG-alkyne-azide-MWCNTs conjugates	Amperometry (HRP/H_2_O_2_/HQ)	TGF-β1	5–200 pg mL^−1^/1.3 pg mL^−1^	Spiked commercial human serum	[41]
SPdCE	A sandwich-type immunoassay involving Btn-DAb and Strep-HRP and the commercial polymeric coating Mix&Go™ for the stable and oriented immobilization of CAb at HOOC-Phe-DWCNTs/SPCEs	Amperometry (HRP/H_2_O_2_/HQ)	IL-1β + TNF-α	IL-1β: 0.5–100 pg mL^−1^/0.38 pg mL^−1^ TNF-α: 1–200 pg mL^−1^/0.85 pg mL^−1^	Spiked commercial human serum and real saliva	[42]
SPCE	Sandwich type immunosensor prepared by immobilizing Btn-cAb onto *p*-ABA-functionalized SPCEs modified with streptavidin and using V-Phe-SWCNT hybrids as nanocarriers of HRP and DAb for amplification purposes	Amperometry (HRP/H_2_O_2_/HQ)	TGF-β1	2.5–1000 pg mL^−1^/0.95 pg mL^−1^	Real human saliva	[43]
SPCE	Sandwich type immunosensor implemented on the surface of HOOC-MBs using Btn-DAb and Strep-AP and Mix&Go polymer for cAb immobilization	Amperometry (HRP/H_2_O_2_/HQ)	TGF-β1	15–3000 pg mL^−1^/10 pg mL^−1^	Spiked human urine and cells supernatants	[44,45]
Au working microelectrodes	Direct immunosensing at CMA-modified Au microelectrodes	EIS ([Fe(CN)_6_]^4−/3−^]	IL-1β + IL-10	1 pg mL^−1^–15 pg mL^−1^	-	[13]
Au working microelectrodes	Direct immunosensing at CMA-modified Au microelectrodes	EIS ([Fe(CN)_6_]^4−/3−^]	TNF-α	1–100 pg mL^−1^/3.1 pg mL^−1^	Human saliva	[14]
Au WE	Sandwich immunosensing using HRP-DAb at CMA-modified Au microelectrodes	Chronoamperometry (HRP/H_2_O_2_/TMB)	TNF-α	1 pg mL^−1^–30 pg mL^−1^/1 pg mL^−1^	Human saliva	[46]
SPCE	Sandwich type immunoassay using Btn-DAb and Strep-HRP implemented on the surface of HOOC-MBs	Amperometry (HRP/H_2_O_2_/HQ)	IL-13Rα2/Cancer	3.9–100 ng mL^−1^/1.2 ng mL^−1^	Lysed and whole cancer cells	[47]
GCE	Sandwich immunosensor prepared at a GCE modified with an AMCs-CTIL composite and using ACP-modified OAMs as nanocarriers of HRP-DAb	ECL and DPV (1-NPP/ACP/HRP) dual detection	IL-6	10 fg mL^−1^–90 ng mL^−1^/0.32 fg mL^−1^	--	[48]
SWCNT-WE	Sandwich immunosensor involving Btn-DAb and Strep-HRP	Amperometry (HRP/H_2_O_2_/HQ)	IL-13	Up to 25 ng mL^−1^/5.4 ng mL^−1^	--	[49]
Au/SPE	Sandwich-based immunosensor involving Btn-DAb and Strep-AP at *p*-ABA grafted Au/SPE	DPV (AP/1-NPP)	TGF-β1	0.05–3.0 ng mL^−1^/10 pg mL^−1^	Spiked commercial human plasma	[11]
Arrays of eight Au microelectrodes fabricated onto needle shaped silicon substrates	Direct immunosensing at Au microelectrodes modified with Sulfo-LC-SPDP and CAb	EIS and DPV ([Fe(CN)_6_]^4−/3−^]	IL-6	--/0.95 pM	Spiked human serum	[50]
Eight Au-SPEs array	Sandwich immunoassays involving HRP-DAb implemented at CMA-Au-SPEs (2D-SPEAu) or Py/Py-COOH/MNPs electrodeposited onto Au-SPEs (3D-SPEAu)	Chronoamperometry (HRP/H_2_O_2_/TMB)	TNF-α	2D-SPEAu and 3D-SPEAu: Up to 15 pg mL^−1^/0.3 pg mL^−1^	Artificial saliva	[51]
GCE	Direct aptasensing at a GCE modified with *p-ABA*, *p-ATP* and AuNPs	EIS ([Fe(CN)_6_]^4−/3−^]	IL-6/Cancer	5 pg mL^−1^–100 ng mL^−1^/1.6 pg mL^−1^	Blood from CRC patients	[52]
GSPE	Direct immunoassay implemented on the surface of ProtG-MBs	EIS ([Fe(CN)_6_]^4−/3−^]	IL-6	1 pg mL^−1^–1 μg mL^−1^/0.3 pg mL^−1^	Spiked human serum	[53]
SPCE	Sandwich immunosensor involving the immobilization of a Btn-CAb onto Strep- modified SPCEs through grafting with *p*-ABA and the use of GQDs/MWCNTs as nanocarrier DAb and HRP molecules	Amperometry (HRP/H_2_O_2_/HQ)	IL-13Rα2/Cancer	2.7–100 ng mL^−1^/0.8 ng mL^−1^	Cellular lysates and extracts of paraffin-embedded tissues from patients diagnosed with colorectal cancer	[54]
SPdCE	Sandwich immunosensors involving the immobilization CAbs onto *p*-ABA-grafted SPCEs and the use of GQDs/MWCNTs as nanocarriers DAb and HRP molecules	Amperometry (HRP/H_2_O_2_/HQ)	IL-13Rα2 + CDH-17/Cancer	IL-13Rα2: 4.92–100 ng mL^−1^/1.4 ng mL^−1^; CDH-17: 0.11–10 ng mL^−1^/0.03 ng mL^−1^	Lysed and whole cancer cells	[55]
SPdCE	Sandwich type immunoassays using Btn-DAb and Strep-HRP implemented on the surface of HOOC-MBs	Amperometry (HRP/H_2_O_2_/HQ)	IL-13Rα2 + E-CDH/Cancer	IL-13Rα2: 3.4–100 ng mL^−1^/1.03 ng mL^−1^; E-CDH: 0.9–25 ng mL^−1^/0.26 ng mL^−1^	Soluble and extracellular fraction of the target biomarkers in serum and paraffined-embedded tissues from CRC patients	[56]
SPCE	Sandwich-based immunosensor involving Btn-DAb and Strep-AP and click chemistry-assisted cAb immobilization by reaction of azide-functionalized MWCNTs and ethynyl-IgG	DPV (AP/1-NPP)	CXCL7/Inflammatory	0.5–600 pg mL^−1^/0.1 pg mL^−1^	Human sera from RA patients	[57]
SPCE	Sandwich-based immunosensor involving Btn-DAb and Strep-HRP at a *p*-ABA grafted SPCE	Amperometry (HRP/H_2_O_2_/HQ)	IFN-γ	2.5–2000 pg mL^−1^/1.6 pg mL^−1^	International Standard and human saliva	[58]
SPCE	Sandwich-based immunosensor involving Btn-DAb and Strep-AP and cAb immobilization onto ethynylated IgG attached to azide-MWCNTs modified electrodes by Cu(I) catalyzed-cycloaddition reaction (electroclick)	DPV (AP/1-NPP)	IL-1β	10–200 pg mL^−1^ and 200–1200 pg mL^−1^/5.2 pg mL^−1^	Spiked human saliva	[59]
ITO	Direct immunosensor at a PPyr-NHS-modified ITO	EIS ([Fe(CN)_6_]^4−/3−^]	IL-6	0.03 pg mL^−1^–22.5 pg mL^−1^/10.2 fg mL^−1^	Human serum	[15]
SPCE	Sandwich immunosensor involving the immobilization CAbs onto *p*-ABA-grafted SPCEs and the use of AuNPs/MWCNTs as nanocarriers DAb and HRP molecules	Amperometry (HRP/H_2_O_2_/HQ)	RANKL/Inflammatory + Cancer	10.4–1000 pg mL^−1^/ 3.1 pg mL^−1^	Human serum from RA and CRC patients	[60]
SPdCE	Sandwich immunosensors involving the immobilization CAbs onto *p*-ABA-grafted SPCEs and the use of AuNPs/MWCNTs as nanocarriers DAb and HRP	Amperometry (HRP/H_2_O_2_/HQ)	RANKL + TNF-α/Cancer	RANKL: 8.6–1000 pg mL^−1^/2.6 pg mL^−1^ TNFα: 9.9–1000 pg mL^−1^/3.0 pg mL^−1^	Human serum from BC patients	[61]

--: information not provided. ACP: acid phosphatase; Ag@Pt: silver@platinum core-shell nanoparticles; 4-APP: 4-aminophenyl phosphate; AMCs: TiO_2_ (anatase) mesocages; AP: alkaline phosphatase; APN: adiponectin; BC: breast cancer; Btn: Biotin C_60_: fullerene; cAb: capture antibody; CDH-17: cadherin-17; CMA: 4-carboxymethyl aryl diazonium; CMC–rGO: carboxymethylcellulose-reduced graphene oxide hybrid; CRC: colorectal cancer; CS: chitosan; CTIL: carboxy-terminated ionic liquid; CXCL7: chemokine C-X-C motif) ligand 7; DAb: detector antibody; DPV: differential pulse voltammetry; DTSP: dithiobis(succinimidyl propionate); E-CDH: E-cadherin; ECL: electrochemiluminescent; EIS: electrochemical impedance spectroscopy; FNAB: 4-fluoro-3-nitro-azidobenzene cross-linker; GCE: glassy carbon electrode; GQDs: graphene quantum dots; GSH-AuNP: glutathione-gold nanoparticles; GSPE: graphite-based screen-printed electrode; HF: heart failure; HNSCC: squamous cell carcinomas of head and neck; HOOC-Phe-DWCNTs: 4-carboxyphenyl-functionalized double-walled carbon nanotubes; HQ: hydroquinone; HRP: horseradish peroxidase; IFN-γ: interferon gamma; IL: ionic liquid; IL-1β: Interleukin-1β; IL-13Rα2: IL-13 receptor α2; ITO: indium tin oxide electrode; ISEs: ion-selective electrodes; LOD: limit of detection; MB: methylene blue; MBs: magnetic microbeads; MWCNTs: multi-walled carbon nanotubes; 1-NP: 1-naphtol; 1-NPP: 1-naphthyl phosphate; OAMs: Octahedral anatase TiO_2_ mesocrystals; p-ABA: p-aminobenzoic acid; PBA: phenyl butyric acid; p-ATP: p-aminothiophenol; PPC: phenyl phosphorylcholine; PPyNPs: polypyrrole nanoparticles; PPyr-NHS: poly(pyrrole N-hydroxy succinimide); RA: rheumatoid arthritis; RANKL: ligand receptor activator nuclear factor-KB; rGO: reduced graphene oxide; TMB: tetramethylbenzidine; SPCE: screen-printed carbon electrode; SPGE: screen-printed graphite electrode; SPE: screen-printed electrode; SPdCE: screen-printed dual carbon electrodes; Strep: streptavidin; SWCNT: single-walled carbon nanotube; TGF-β1: Transforming Growth Factor β1 cytokine; TNF-α: factor necrosis tumor α; V-Phe-SWCNT: viologen-functionalized SWCNT; WE: working electrode.

## Data Availability

Data sharing not applicable.
